# Research avenues for amplifying Indigenous radio

**DOI:** 10.12688/openreseurope.14020.1

**Published:** 2022-03-04

**Authors:** Katie Moylan

**Affiliations:** 1 English, University of Texas at Arlington, Arlington, Texas, 76019, USA; 2 Media, Communication and Sociology, University of Leicester, Leicester, Leics, LE1 7RH, UK

**Keywords:** Indigenous radio, community-building, mapping, Indigenous theory, production practice, radio content, radio, mapping, COVID-19

## Abstract

In this article, I discuss Indigenous radio’s ongoing importance for tribal communities in the US from my perspective as a settler scholar, drawing on multifaceted research into Indigenous radio’s programme content and production practices before and during the pandemic. For this research, ‘Indigenous radio’ refers to radio produced, managed, presented and/ or owned by tribal communities. Other terms in use to describe Indigenous radio include Native American, Indian, or tribal radio, demonstrating that there is not a single universalising term and reflecting a diversity in tribal cultures, languages and practices more generally. Building on this understanding of the inherent diversity of Indigenous radio, I describe the ways in which my overarching research project investigates Indigenous radio holistically, via critical outputs combining a literature review of Indigenous theoretical approaches, an online interactive map of tribal stations and in-depth case studies of tribal stations. Through these, I explore community-building practices of Indigenous radio as produced through what Indigenous theorists Glen Coulthard and Leanne Betasamosake Simpson (2016) term grounded normativity. Building on this avenue of exploration, I suggest the place-based values embedded in Indigenous radio production practices and content can function as everyday acts of resurgence, following Jeff Corntassel’s (2012) conceptualisation of ways in which Indigenous resurgence can reinforce a project of decolonisation. To exemplify and situate these arguments, I draw on examples of radio production and practitioner insights from selected tribal stations embodying diverse tribal production practices and content, before turning to focus on pandemic practices in Indigenous radio. When the pandemic emerged, my research focus necessarily widened to include and examine COVID-related practices and programming in tribal radio, enabling reflection on these in the context of a paradigm shift in which the value of tribal radio's community-building work has become acute.

## Plain language summary

In this article, I discuss my research into Indigenous radio and describe its importance as a both a communication tool and a form of community-building for the tribal peoples who produce it and whose communities it serves. This article explains the value of Indigenous radio for tribal community-building and self-determination in US geographical contexts before and during the pandemic. The article identifies and defines appropriate approaches from Indigenous scholars for studying Indigenous radio content and production practices. This is followed by analysis of examples of Indigenous on-air community-building, in which I identify communicative support strategies in tribal radio during the pandemic. From the start of the pandemic, tribal radio stations provided essential information and community support on-air, building on established trust of their tribal listening communities and further demonstrating the cultural and social value of locally produced Indigenous radio content. I conclude the article by discussing the ongoing importance of Indigenous radio in these crisis times for providing community and infrastructural support, even as Indigenous peoples’ struggles for self-determination are increasingly recognised at wider political registers.

## Introduction: What is Indigenous radio?

There are estimated to be between 50 and 70 Indigenous-owned and managed radio stations on reservations in the 50 US states, and Loris Taylor, CEO of Native Public Media puts the number at 60. ^1^ This station provision remains fundamentally under-representative of the diversity of Indigenous communities across the US given there are (at time of writing) 574 federally recognised Tribal Nations (variously described as bands, communities, nations, pueblos, tribes, and Native villages) as well as state recognised tribes, all defined as Indian Nations by the National Congress of American Indians (NCAI), alongside further Indigenous communities currently not officially recognised by the NCAI. ^2^ In this article, I identify and consider the cultural and societal importance of Indigenous radio for the communities served and explore ways in which values embedded in radio production emerge from what Indigenous theorists Glen Coulthard and Leanne Betasamosake Simpson (2016) term *grounded normativity*. An Indigenous concept, *grounded normativity* describes how specificities of place-based practices central to Indigenous everyday life shape and reinforce distinct tribal identity. To expand on this exploration, I suggest that place-based values embedded in Indigenous radio production practices and content can function as everyday *acts of resurgence*, following Jeff Corntassel’s (2012) conceptualisation of ways in which Indigenous resurgence reinforces decolonising processes. To situate how these theoretical arguments frame my radio research, I begin below by describing the multifaceted research design I developed to allow me to more thoroughly examine and explore diverse tribal production practices and content components. When the pandemic began to take hold, my holistic set of research approaches also enabled me to widen my research focus to incorporate and examine COVID-related practices and programming, and to reflect on these in the context of a fundamental paradigm shift affecting everyday life for all of us; radio practitioners and academics alike.

In this multistranded research, ‘Indigenous radio’ refers to radio produced, managed, presented and/or owned by tribal communities, which foregrounds locally produced Indigenous community programming. In research conversations and in Indigenous radio content, practitioners and other tribal community members use different terms, including Native American, Indians, Native radio, tribal radio and Indigenous, as well as tribal names such as Chickasaw, Hualapai, Hopi, Pala, Mountain Ute and Southern Ute. This diversity in everyday uses of pan-Indigenous terminology reflects a deep diversity in tribal cultures, languages and practices, further demonstrating that there is not a single universalising term agreed upon by all. Instead, therefore, I move between the terms Native American, Indigenous and tribal in my research but foreground specific tribe names where possible. In the US, many, even most Indigenous stations broadcast via a community radio or Low Power (LP) license, meaning that some are under-funded and must continually raise money to ensure the ongoing smooth running of the station. Other Indigenous stations are supported by casino revenue on those reservations which host casinos. An alternative license category is the Tribal Priority license, which provides further resources, although these licenses are fewer and competitive to acquire. Most tribal stations therefore remain under-resourced in material terms, which can make them especially vulnerable to infrastructural and technology crises. Jana Wilbricht explains the particular value of radio in relation to everyday life for many Indigenous communities, observing that

Radio is accessible even to households without electricity at very low cost, does not require literacy, provides programming in the local Indigenous language, and blends with rural lifestyles, e.g. in terms of requiring less attention than television and print media, which do not allow for multitasking and information consumption in the outdoors as readily as radio

                                                                                                                                                                                                                          ( Wilbricht, 2019: 53)

Also writing about Indigenous radio, Carlos Jimenez argues that the ‘community-radio model suits low-income communities that have an urgent need to be informed and connected’ ( Jimenez, 2019: 266). Many reservation-based radio stations have an Emergency Response capacity, meaning that for people living on the reservation these stations are often the first source of information in local crisis situations, including forest fires and extreme weather. At the same time, despite substantial material and structural commonalities, tribal stations facilitate and enable self-representation of their target communities in distinct and diverse ways based on the needs of their local listenership.

To clearly and comprehensively introduce and situate my multifaceted research explorations, the first section of this article explains my evolving research approaches, incorporating the development of an online map of Indigenous radio stations which has become an (unplanned) standalone output. The second section outlines particularities of place and tribal specificities of five Indigenous stations which comprised case studies, to compare and demonstrate distinct and differing characteristics of Indigenous radio and explain how these stations enable Indigenous self-representation and self-determination. This section draws on Indigenous radio practitioner perspectives to illuminate the value/s of these tribal radio stations for their communities and unpacks how Indigenous radio practices can be considered as embodying decolonising place-based acts of resurgence. The third section identifies ways in which tribal stations have continued their community-building work during the current pandemic to provide ongoing emotional and infrastructural support across multiple communicative and structural registers during these unsettled crisis times. The final section then returns to (re-)consider my overarching research design and reflect on how my research assemblage of established academic approaches (incorporating an Indigenous literature review, mapping of the field and determination of selection criteria), alongside Indigenous research approaches of relationality and reciprocity, has continually enabled in-depth radio research during these simultaneously ‘unusual and familiar times’ ( Weaver, 2020). I suggest that this multifaceted research design, which expanded and shifted along with the advent of the pandemic and its consequent effects on my planned onsite fieldwork, has allowed for these seismic societal shifts through incorporating recognition that community-facing research is always in process and is fundamentally built on relationality, reciprocity and reflexivity.

## Mapping Indigenous radio: Mapping as method

My research focus on radio’s medium-specific and expansive capacities for Indigenous self-determination emerged from my earlier explorations ( Moylan, 2018; Moylan, 2019) into ways in which radio technology has historically enabled a capacity for ‘community-building’ (described by Hartley, 2000, see also Hendy, 2000), incorporating the potential to facilitate a collective sense of belonging through shared programming produced by and for the community. Through such grassroots programming, radio activates its inherent capacity for *reproducing locality*, strengthening a sense of belonging for communities connected by both cultural affiliation and geography. I further suggest that the radio medium particularly lends itself to Indigenous cultural expression because as an aural form it emphasizes capacities for oral storytelling and particular uses of cadence and delivery.

In approaching and developing this multifaceted and multi-sited research, I found myself at the start of my Fellowship working in two distinct and separate directions. In growing recognition of the multifaceted significance of Indigenous radio as a community-led, alternative form of media, I wanted to expand on this by developing a comprehensive list of radio produced by Indigenous communities. While I was already familiar with several Indigenous radio stations from past fieldwork, I considered that I needed to substantially investigate the scope of Indigenous radio provision across the US, which meant temporarily stepping back from the in-depth explorations I had previously undertaken. There wasn’t a standalone map exclusively detailing Native American owned and managed radio stations, although the Native Voice 1 website (see https://www.nv1.org/) includes a map of radio stations broadcasting tribal content incorporating National Public Radio affiliates, which are not tribally owned or managed. I began to systematically search for and identify Indigenous-owned and managed radio stations and standalone Indigenous radio shows produced at other, non-Indigenous stations. I did this by using different search terms reflecting the different descriptions in use for Indigenous radio in the US: ‘American Indian radio’, ‘Native radio’ and ‘Native American radio’, ‘Indigenous radio’ and ‘tribal radio’, then going through the results to confirm tribal affiliations and locations. Initial information on radio stations was also gathered from the Native American Journalists Association and Native Public Media and the digital media platform Indian Country Today. Additional radio station information was gathered in conversation with practitioners in Indigenous radio. Once I discovered a new station I cross-referenced its online presence; many tribal stations have their own webpage but some are accessed from their tribe’s main page (for example, KPYT FM, the radio station of the Pascua Yaqui tribe, has a webpage on the tribe’s main site at https://www.pascuayaqui-nsn.gov/). Additionally, many tribal stations have a Facebook page. Finally, I also checked for streaming services, which again vary (and also depend on what country or jurisdiction you are trying to access the streaming content from.)

My map of Indigenous stations thus began as a research tool enabling me to identify the scale of tribal radio in US geographical contexts, which then expanded into an in-progress informational resource as I started sharing it with tribal radio practitioners for their feedback as to its usefulness and for any corrections for accuracy and improvements. The map therefore serves layered functions: it provides a geographical overview of Indigenous radio stations in the US and incorporates links to each station’s webpage. Connecting the map user with the tribal stations represented enables them to find out more about each station and also, crucially, to listen live. Most of the stations included on the map incorporate livestreaming online, expanding their broadcast reach beyond that of the antenna. Incorporating streaming capacities where the station has enabled these is central to the map’s overall function; including a streaming link per station via the map provides users with immediate access to live Indigenous radio, allowing them to hear the diversity in programming and content. By focusing solely on local tribal radio, this map demonstrates the ongoing resilience of Indigenous community radio across the US.

As my lists of stations and standalone shows became longer, it made sense to transfer these to a visual form so the scale could immediately be apparent, which in turn shifted this from a scoping exercise to a standalone map, albeit still open-ended and in process. I recognise that creating a map carries significance beyond producing a seemingly objective representation of existing phenomena. In settler colonial contexts in particular, physically mapping Indigenous spaces and places builds on a historical practice of colonising space, both symbolically and by enabling physical encroachment and invasion, usually through violence. Maps have comprised a technology of colonialism, making possible settler colonial practices. In recognition of the historical and political significance of mapping as process and as product, I consider this map as a text in its own right and examine the ways in which it has the capacity to contribute to (or to derail) a decolonising project. Dallas Hunt and Shaun A. Stevenson examine and explore Indigenous counter-mapping, or re-mapping practices, and argue persuasively that while these can embody and fulfill a decolonizing purpose, they may also simultaneously incorporate elements of existing mapping technologies, including digital technologies, which potentially embed them into a dominant settler colonial paradigm. As they explain, ‘the very language of mapping may itself perpetuate a kind of colonial incursion into particular Indigenous conceptions of place and space’ ( Hunt & Stevenson, 2017: 377).

Further, situating and studying Indigenous radio ‘in the US’ must incorporate recognition that the ‘United States’ is itself a product of and embodies ongoing settler colonialism of the hundreds of Indigenous tribal nations and communities which are the first peoples of North America. All maps created through settler colonialism can be understood as comprising a historicised reframing of place in accordance to their colonial governance. In the same way, in their deployment of legacy broadcasting and streaming technologies (especially via platforms such as Facebook), radio stations are themselves part of the technological but also discursive dominant infrastructure which reinforces settled colonial logics in the US. Against this, however, I argue that tribally-specific production practices and content functions as an alternative to these dominant discourses, in turn decolonising the airwaves (in the sky and on the spectrum). This recognition led me to create a title for the map: 'Sovereignty of the Air: Amplifying Indigenous Radio', to reflexively mark it as a textual as well as informational object. The map can be found here https://tinyurl.com/MappingIndigenousRadio ( Moylan, 2022).

While I developed this map specifically to represent Indigenous radio in US geographical areas, my next step is to expand its coverage to incorporate Indigenous radio elsewhere in the world—not least because the lands represented by many Indigenous stations have been inhabited by tribal communities for centuries, in some places, meaning in turn that designations such as ‘the US’ to describe these spaces are fundamentally irrelevant. The US, Canada, Australia, and New Zealand are all settler colonial names describing land that was already lived on.

Michelle M. Jacob asserts that ‘indigenous decolonisation is about reclaiming traditions, in addition to moving forward in the complex social, political, and economic realities colonisation brought to our people and homelands’ ( Jacob, 2013: 6) and I argue that Indigenous radio’s considerable capacities for community-building contributes to this movement. As I explain further below, Indigenous radio’s value lies in multiple areas and activities: in enabling tribally-specific articulation and expression, in turn strengthening tribal self-determination; in its inscription of Indigenous voices offering an alternative to a totalising, reductive mainstream set of on-air discourses which continue to privilege normative white middle class subjectivities; in its dissemination of diverse and distinctive Indigenous music, language and stories, to preserve and amplify these by regularly playing them on air, enlarging and enriching the range of available music, languages, stories and voices on air and online. To examine these diverse cultural and linguistic articulations across tribal stations, in addition to mapping stations I mined tribal radio station schedules online, exploring these to identify local talk and music programming by and for a station’s tribal community (links to selected radio schedules can be found in the *underlying data*).

Radio schedules are themselves a rich resource, as they provide a portrait of different types of programming along with the time of day/night these are broadcast, which can indicate a show’s popularity amongst its community listenership. Both a show’s topic (discussion or music, traditionally tribal or contemporary) and its scheduled time slot are relevant to practitioner expectations of listener requirements. Music programming is most often scheduled in evenings and/or weekends, with each evening offering a different music genre, while the ‘drivetime’ slots of 9am and 4 – 6pm often foreground talk and information provision. Many schedules also reveal a balance of local and syndicated programming, usually incorporating a mixture between locally-produced tribal programming and nationally syndicated Native American programming alongside programming from US National Public Radio (NPR), Pacifica or other non-profit broadcasting networks. Additionally, I mapped Indigenous shows produced at other radio stations—usually community or NPR affiliate stations in urban areas, featuring programming by and for intertribal communities which had grown since historical relocation.

My research design thus expanded as my research objects did, to allow for multifaceted ways of exploring, situating and understanding diverse content and station structures. Shawn Wilson argues that in reaction to unasked-for research conducted in their tribal communities, an Indigenous approach to research must embody an encompassing, holistic framework in which ‘the research methodology needs to incorporate their cosmology, worldview, epistemology and ethical beliefs. An Indigenous research paradigm needs to be followed through all stages of research’ ( Wilson, 2008: 15). In widening my research parameters and consequently analytical approaches, I came closer to developing a more holistic critical framework.

Alongside these mapping practices, I developed a literature review of Indigenous critical theory to more appropriately situate and explain Indigenous cultural production practices (the literature review can be found in *underlying data*). As this was a new area of scholarly enquiry for me I began with a recommendation from Dr Les Riding-In at the University of Texas in Arlington, who was part of a research cohort established to help me navigate my research at the University in my first Fellowship year. Dr Riding-In explained the importance of concepts of sovereignty and self-determination for Indigenous approaches (see Lomawaima & McCarty, 2002) and shared several articles situating these. Another formative article, ‘Decolonization is not a metaphor’ by Indigenous scholar Eve Tuck and K. Wayne Yang (2012) deepened my critical understanding of the necessary work of decolonization. I developed an Indigenous literature review by searching further for articles, books and book chapters exploring these and other Indigenous concepts, in part by mining the Bibliographies of my growing list of sources and by foregrounding work by Indigenous scholars. I soon recognised that journals such as *Decolonization: Indigeneity, Education & Society* and the *ACME Journal for Critical Geographies* regularly published Indigenous critical scholarship, while the University of Arizona, the University of Nebraska and University of Toronto regularly published Indigenous academic books.

I have found that Indigenous critical scholarship is often multi-disciplinary, incorporating concepts from disciplines including communication, education, geography, history, law, literature and musicology. Storytelling and reflection are modalities through which Indigenous theory is often generated and expressed, emerging from and rooted within particularities of distinct tribal practices of the author/s (see Betasamosake Simpson, 2017; Tuhiwai-Smith, 1999 and Wilson, 2008, in particular).

My onsite fieldwork at tribal stations was informed by Indigenous concepts of relationality and reciprocity, to more precisely explore the significance Indigenous radio practitioners give to their radio work, I encouraged open-ended conversations which enable me to, as described by Rosalind Gill and Andy Pratt, ‘engage with the specificities of different…workplaces and locations, and attend to the meanings that workers themselves give to their labour’ ( Gill & Pratt, 2008: 21). I was fortunate to be able to visit three stations: KCNP, KUYI and KPRI, before the first lockdown in March 2020. After then, I followed up with practitioners by phone or on Zoom to develop in-depth explorations into the tribally distinct ways each station interacted with, represented and supported its surrounding communities through multiple forms of community-building. (These in-depth studies will be published separately; see Moylan, 2022). Our research conversations, whether in-person over the phone or online, developed around practitioners’ reflections on their community-facing radio practices and insights into their significance for local community-building. In these unstructured discussions, we found that practitioners describe diverse production practices and connect these back to community representation in their own terms.

Indigenous community-produced radio, while embodied in multiple, distinct and myriad forms, at the same time can share experiential and material commonalities. Tribal radio station spaces function as sites for localised production practices but also often serve as a physical meeting point for local tribal communities. Therefore in analysing station structures alongside situated production, the connections between production practice and the space stations provide for this coalesce to situate these community-facing practices within place-based *grounded normativity* particular to the region and its tribal community. The Indigenous concept of grounded normativity is defined by Glen Coulthard and Leanne Betasamosake Simpson as a place-based situatedness which shapes ‘Indigenous place-based practices and associated forms of knowledge’ ( Coulthard & Betasamosake Simpson, 2016: 254). If practitioner perspectives are shaped by ‘the meanings which people give to their experience’, that is, how practitioners consider and define their production work, drawing on insights from these conversations can foreground ‘the meanings cultural workers themselves give to their life and work’ ( Gill & Pratt, 2008: 19). In turn, then, practitioner insights can strengthen recognition that the value and meaning of Indigenous radio is located in both content and production practices as modes of community-building. Gathering practitioner *knowledges* alongside developing a multidisciplinary literature review and creating an Indigenous radio map has provided a multifaceted framework which allowed for—even foregrounded—more nuanced and thorough exploration of the diverse particularities of place-based community-building in Indigenous radio stations.

## Embodying place-based practices in local tribal programming

Through the multifaceted approaches unpacked above, and by building on existing practitioner relationships, my research evolved to incorporate in-depth studies of five tribal stations (as well as of a standalone radio show, *Beyond Bows and Arrows*, broadcast in Dallas; see Moylan, unpublished data.) The stations in these studies comprise KWLP (Hualapai tribe), KUYI (Hopi tribe), KPRI (Pala Band of Mission Indians), KSUT (Ute Mountain Ute and Southern Ute tribes) and KCNP (Chickasaw peoples). The five stations differ from each other in their infrastructure, surrounding geography and the communities they serve and represent. These differences in turn demonstrate this research’s underpinning recognition of the tribally-specific particularities which characterise each station’s programming and practices. Yet at the same time commonalities across programming sit alongside structural and material commonalities. Many Indigenous stations in the US broadcast the syndicated show *Native America Calling*, which is produced and streamed by the Native Voice 1 (NV1) network in New Mexico, alongside locally-produced tribal and non-Native programming. Additionally, and crucially, many Indigenous stations, including these five, can be accessed through both analogue broadcasting and online streaming. Their programming can be heard locally via FM radio frequencies but also accessed online from anywhere in the world.

In northwest Arizona, an hour’s drive from the closest city, Kingman, and broadcasting locally on 100.9FM, KWLP serves the Hualapai people in the town of Peach Springs on the edge of the Grand Canyon:

The station broadcasts live from the Hualapai Indian Reservation which encompasses a million acres along 108 miles of the Colorado River Grand Canyon--Home of the Hualapai River Runners and the Grand Canyon West Skywalk. The Hwal`bay call this middle river corridor "Hakataya" or "the backbone of the river" ^3^

KWLP’s building includes a reception office, manager’s office and studio spaces. The Hualapai station’s schedule combines a mixture of talk and music programming across multiple genres, including bluegrass, country, gospel, reggae, rock and urban, as well as a range of locally popular Indigenous music, from birdsong to ‘country rez’ (so described by one KWLP practitioner) to powwow songs. As is usual in community-led radio, each show’s volunteer presenter is often also the producer and thus responsible for the music chosen. Most of KWLP’s on-air practitioners are women, several of whom have been producing and presenting their shows for years. One long-running local show is *Native Noon Hour*, presented by established practitioner Wanda ‘Wanda J’ Quasula on Mondays through Fridays at 12noon. *Native Noon Hour* exclusively plays Indigenous artists, as the show page states: 'only native artists: local and national, traditional and contemporary' ^4^ alongside local content such as weather and community updates. Each show includes regional powwow and Indian rodeo schedule updates, alongside the Hualapai word and phrase of the day. KWLP’s eclectic weekend schedule includes two hours of children’s programming on Sunday evenings, featuring stories for children. *Tales of Wisdom* is a rebroadcast podcast which incorporates stories from diverse cultures, while *Read on Hualapai* is produced by KWLP and focuses on Hualapai storytelling. ^5^

Across Arizona to the northeast, KUYI broadcasts on 88.1FM and 89.1FM and serves Hopi and Tewa communities on the Hopi reservation. These lands are encircled in turn by Navajo, or Diné lands, meaning that KUYI is listened to by Hopi, Tewa and Diné alike, as well as anyone else within range of their strong broadcast signal which can be picked up from Flagstaff to Winslow and is produced via two antennas, with the original signal bolstered by a newer LP antenna. Similarly to KWLP, KUYI also features an eclectic schedule combining talk and diverse music programming and (as with KWLP and most other tribal stations) and most programmes are again produced and presented by local tribal community volunteers. The weekday schedule incorporates locally produced programmes such as *The Hopi Teen Show* and health information show *House Calls* alongside syndicated programmes from NV1 and NPR. On weekdays music programming combines tribal music in the mornings on the *Tatawi* show and Indigenous and Oldies shows in the afternoons. Evenings are dedicated to music genres including blues, Cajun and Zydeco, reggae and rock. KUYI’s weekend programming similarly moves between talk and music radio, featuring a mixture of traditional powwow music and contemporary music shows alongside talk programming from NV1 and NPR. ^6^

In southern California and owned and operated by the Pala Band of Mission Indians, KPRI serves Pala and non-Native communities on Pala lands, broadcasting on 93.1FM and also widely known as Rez Radio. The schedule combines local and syndicated programming, which is a mixture of talk and music. *Pala Today News* broadcasts Mondays through Fridays at 12noon and provides in-depth coverage of local news. KPRI’s music programming reflects Pala community preferences and incorporates genres from reggae to rock to hiphop to oldies. All local music shows are produced and presented by volunteers from the surrounding community in Pala. *Songs of the Southwest* on Saturday mornings plays local birdsong and other traditional music from the region. *Pala Life Past and Present* provides historical insights into local tribal traditions and cultural events, broadcasting Mondays directly after *Pala Today News*. Eric Ortega, who produces and presents both shows, summarises KPRI’s community-building work in a Pala saying: ‘Hiachuwenesh pe’ ‘achivet, Miyaxwanesh ‘ichaam ‘emáy, Nanvaxyaxwenesh pe’chu’ muchika.’ ^7^ Translated into English, this is: ‘Learn from the past, live well in the present, prepare for the future.’ Ortega expands on the motto’s meaning, saying

You know, this radio station was actually built because of the fires, and [need for] communication, so in a way this radio station is an example of us remembering the past as far as the dangers involved, and with the successes we’re having with the radio station as far as the awards we’re getting and stuff like that, is causing us to live well in the present. At the same time, we’re still always practicing and preparing for the future, so the phrase was actually pretty cool as far as this radio station’s concerned. It kind of became our unofficial motto.

                                                                                                                                                                                                                          (Interview with Eric Ortega at KPRI, 12 March 2020)

As Ortega observes, this motto reinforces recognition of the ongoing connectedness between KPRI’s history as a local emergency information source for wildfires and other crisis events; its current critical successes in which its on-air preservation is celebrated, and its ongoing plans to continue with its unique programming into the future.

Located in Ignacio, near Four Corners, Colorado, KSUT was created as a tribal station which today serves the communities of the Southern Ute, Ute Mountain Ute, Jicarilla Apache, and Navajo, or Diné, lands. KSUT broadcasts from three local frequencies: 91.3 FM in Ignacio, 100.9 FM in Towaoc, both in Colorado; and 89.7 FM in Farmington, New Mexico ^8^. Additionally and unusually, KSUT also incorporates a separate National Public Broadcasting (NPR) programming stream, so that the station is a hybrid of tribal and NPR affiliate programming. The Tribal Radio schedule features the *Tribal Radio Morning Show* Mondays through Fridays from 8am to 11am, alongside a combination of locally produced and syndicated Indigenous programming. KSUT Tribal Radio manager Sheila Nanaeto explains the importance of intertribal Indigenous representation on-air as she describes the station’s history in the Four Corners area of Colorado, which also borders Utah, Arizona and New Mexico and therefore is home to several tribal communities in the region:

We’re a tribal radio station, we started out as a tribal radio station, and now we’ve grown into a Four Corners public radio station, serving two distinct communities…
  We want to serve the communities in our area, and we know there are other tribal stations but they’re very specific to their tribe, and we’re trying to include everybody because our reach is so big now, from when we first started.

                                                                                                                                                                                                                          (Interview with Sheila Nanaeto, 18 June 2020)

In addition to being home to multiple communities, the surrounding region hosts several other tribal stations, as Nanaeto observes and as I found when I visited; driving through north-east Arizona and into New Mexico enroute to the fieldwork visit at KUYI, the car radio picked up both KUYI (Hopi) and KYAT (Navajo/Diné) stations. Other stations in the region are KTNN and KNDN, both serving local Navajo/Diné communities.

In Oklahoma, Chickasaw radio station KCNP broadcasts on four frequencies across southern Oklahoma: 89.5 FM in Ada, 89.3 FM from Dickson/Ardmore, 97.3 FM from the Connerville/Tishomingo area and 104.5 FM from Wynnewood/Pauls Valley ^9^. The schedule contains a diverse mixture of locally produced and syndicated programming which combines music and talk radio, and which reflects the diverse nature of the communities—both Indigenous and non-Native—served and represented by KCNP’s programming. Station manager Brian Brashier explains that in determining KCNP’s schedule, they had to account for diverse preferences across the communities they serve and observes that ‘the Chickasaw people are not so homogenous where they’re all just one thing; they’re not.’ Through KCNP’s community updates on cultural events, he suggests that ‘people are engaging in their culture, and they never had a chance to do that before, and that was through us’ (interview with Brian Brashier, KCNP, 14 November 2019). Even more crucial for local communities (again as with other tribal stations) is KCNP’s emergency weather coverage. Brashier describes the importance of KCNP’s weather coverage during the storms and tornados which are common events in Oklahoma.

A lot of people here have a storm shelter because of the tornados and the severe weather. Their TVs won’t work in there but the radio will, in most cases. It’s easier to keep your radio batteries up than your cellphone batteries….
  We’re way in advance trying to get people ready. On the day of, we keep reminding them: your power could go off tonight. You want to stay tuned to us because we’ll tell you when it’s safe to come out of your shelter: that’s the other thing you don’t hear on other media.

                                                                                                                                                                                                                          (Brashier interview, 2019)

I suggest the place-based programme production practices outlined above reinforce tribally-specific community-building as produced by each station’s *grounded normativity*, enacted within place-based practices located in and shaped by the surrounding geography but also by localised tribal practices which have emerged from these lands. Comprised of these everyday place-based articulations of tribal specificity, Indigenous radio content reinforces a shared sense of local community within what Corntassel terms ‘daily acts of *renewal*’. These acts of renewal take culturally specific forms coalescing in ongoing acts of resurgence, which can incorporate practices such as ‘prayer, speaking your language, honoring your ancestors, etc’ ( Corntassel, 2012: 89). KPRI’s station manager John Fox explains how they reinforce the station’s ‘Indianness’ through local, unique programming:

We need to be different, and unusual… in terms of this particular place, and I would think with any tribally-owned station, is to embrace your local culture, so when there is a birdsong gathering, or the Cultural Weekend that they have every first weekend of May, when they invite tribes from all over the southwest to come and dance and sing and show their own particular style, then we find it important to broadcast all those things live. That’s basically what gives Rez Radio its Indianness.

                                                                                                                                                                                                                          (Interview with John Fox at KPRI, 12 March 2020)

Such acts of resurgence in turn embody elements of ‘the spiritual, cultural, economic, social and political scope of the [decolonizing] struggle’ ( Corntassel, 2012: 88), which contribute to the overarching work of decolonisation in the ongoing context of settler colonialism in the US while amplifying a diverse range of distinctive tribal cultural expression on-air.

## Broadcasting local community support during the pandemic

From mid-March 2020, the advent of the pandemic immediately and for many practitioners fundamentally altered broadcast practices across all radio sectors, including commercial and public service radio as well as community stations. Radio presenters and producers everywhere have had to radically adjust both their work practices (many necessarily moving to remote broadcasting and programme production from home) and the components of show content, newly reconsidered to reflect the concerns of an anxious and mainly quarantined listenership. The importance of broadcast radio for information provision and community-building has become much more urgent everywhere during the COVID-19 crisis. This shift additionally reflects an emerging paradigm in which the importance of *localised* broadcasting has acquired a stark new significance given that cities, regions and states around the world have experienced the pandemic and managed protocols and responses in very different ways. The need for reliable local coverage and reporting has become acute.

As COVID-19 took hold across the US, for many Indigenous communities, particularly those located in lands otherwise isolated from services available in towns or cities, tribal stations have served as the first and main source of local COVID-19 news, updates and information. More generally, at the same time as most US states faltered in providing solid guidance and/or protections for their constituents, Indigenous strategies on the ground embodied viable approaches for negotiating the pandemic and its fallout. As Hilary Weaver explains, ‘Exercising their inherent sovereignty, Indigenous Peoples have implemented sometimes parallel and sometimes distinct responses to the pandemic’ ( Weaver, 2020: 13). Existing tribal sovereignty has enabled autonomous structural responses from multiple tribes, often in advance of US state responses and in places challenging these where state mandates are unsafe or non-existent.

In south Oklahoma, the Chickasaw tribal community implemented measures promptly, as KCNP manager Brian Brashier explains:

One of the first things that happened, was for the protection of our citizens, guests, and employees, carefully and logically shutting things down. It was pretty fast; it was really fast in fact.

                                                                                                                                                                                                                          (Interview with Brian Brashier, KCNP, 22 June 2021)

Throughout the pandemic the Chickasaw have facilitated a wide range of infrastructural activities to help and support local community members—Chickasaw and non-Indigenous alike—providing everything from food supplies to vaccination centres. In northwest Arizona, the Hualapai tribe similarly implemented their lockdown in advance of Arizona’s mandate and maintained strong protocols to keep tribal community members safe, as KWLP manager Terri Hutchens observes:

The tribe was very proactive in imposing precautions and restrictions, and they kept their numbers—positives and deaths—down to an amazing minimum, compared to the surrounding non-Native communities.

                                                                                                                                                                                                                          (Interview with Terri Hutchens, KWLP, 23 June 2021)

From mid-March 2020, tribal stations produced and disseminated local updates and Public Service Announcements (PSAs) about the developing pandemic, reflecting the particularities of the communities they serve. The Chickasaw network, broadcasting from KCNP in Ada, Oklahoma and three other stations in southern Oklahoma, developed precautionary protocols for in-studio use early. These included a no guest rule in the station studios and mask wearing by all radio DJs unless alone in the studio. Brashier observes:

When we started seeing this, I wrote up a list of protocols based on what I read from the CDC and others, sent it to my staff and said “can everyone live with this?” and they all said “yes.” So before just about anyone else shut it down, we shut it down.

                                                                                                                                                                                                                          (Brashier interview, 2021)

Once physical safety protocols were in place Chickasaw radio remained on air throughout the pandemic and the number of on-air interviews actually increased by utilizing remote technologies such as Zoom. ^10^

KWLP served as the primary source of pandemic-related news and information from the pandemic’s start, as Hutchens explains:

We did a lot. We actually became a hub, the primary hub for disseminating information for the community about what the tribe was doing. We broadcast all the Tribal Council meetings live and they had about three a week. Two a week that were specifically geared towards COVID intervention and issues; funding and getting resources to the community.

                                                                                                                                                                                                                          (Hutchens interview)

The Hualapai tribal lands are isolated from municipal infrastructures and can experience electricity blackouts and brownouts, which affect communication networks when the power goes out. Consequently, the community has developed technical workarounds to ensure communication, but these had drawbacks compared to KWLP's reach:

Because the newspaper, the community uses flyers posted around the community, but of course they couldn’t do that and they couldn’t see them if they were there, and the newspaper only comes out every two weeks, so sometimes when it would come out things would already have changed.

                                                                                                                                                                                                                          (Hutchens interview)

In recognition that KWLP is the central source for local news and information for the geographical and tribal community, Hutchens describes how the station also arranged for distribution of portable radios to community members:

We gave about 50 radios that had handcrank generators so they didn’t have to go get batteries. And we do have power outages a lot here, brownouts and blackouts, particularly during monsoon and then the severe weather in the winter, so people do get stranded. And if you can’t run to the store in Kingman to get more batteries because of COVID lockdowns [the radios will still work]

                                                                                                                                                                                                                          (Hutchens interview)

Writing about Indigenous responses to the pandemic, Hilary Weaver observes that ‘[w]ith full awareness that many Indigenous people and communities lack adequate access to on-line infrastructure, strategies were developed to meet the needs of different contexts’ ( Weaver, 2020: 17). Such localised strategies, exemplified by KWLP’s distribution of handcrank radios but also by ongoing communicative strategies on tribal radio, have roots in past Indigenous experiences of genocide, emerging from the systematic violence of settler colonialism. Localised tribal practices were historically developed by Indigenous communities across the US, first created to combat crises created by settler colonial practices. These practices of resistance in turn informed tribal community-building, as Indigenous historian Roxanne Dunbar-Ortiz explains: ‘Today’s Indigenous nations and communities are societies formed by their resistance to colonialism, through which they have carried their practices and histories’ ( Dunbar-Ortiz, 2014: 7). Today, enduring tribal practices enable and sustain what Linda Tuhiwai-Smith identifies as Indigenous imperatives of ‘the survival of peoples, cultures and languages’ ( Tuhiwai-Smith, 1999: 142). In the face of another potential genocide, such approaches and knowledges, determined by the particularities of place and forged through resilience, continue alongside protocols (or a lack thereof) mandated by top-down state directives too often informed by economic rather than community care imperatives.

## ‘By being apart, we’re now closer’: Exploring Indigenous radio into the future

The advent of the pandemic and the many ways in which this has transformed ways of working—for myself and for tribal radio practitioners I’ve been in conversation with—has shaped the progress of this research in multiple ways. Like everyone else, I started to approach all communication with more care, to foreground compassion in my emails, online and on the phone, and to think carefully about how best to reach out to people. With two scheduled onsite visits necessarily cancelled in Spring 2020 and further planned visits halted, I contacted practitioners primarily by email, following up with phone calls and via Zoom when possible. Most radio practitioners at Indigenous radio stations were firefighting across various registers, developing and managing updates about the pandemic and related activities such as available testing, elder shopping hours, lockdown guidelines and cancelled graduations and other events. At the same time, fires were ravaging parts of the US southwest, including regions covered by stations I was in touch with, adding another layer of everyday emergency information provision which urgently needed to be regularly updated.

As paradigm shifts increased everywhere in localised ways, the Indigenous radio production practices which form the central focus of my scheduled multi-sited research also fundamentally altered. As with almost all broadcast radio, most stations began producing and broadcasting programmes and news updates remotely. My methods needed to change to reflect these changes and to represent these on their own terms. Fortunately, the expansive scope of my two-year Fellowship enabled me to develop new ways of working—of thinking, exploring, discussing and researching—as pandemic-related events continued to unfold in distinctly different ways for the five stations included in my research. From the start of my Fellowship, the scope provided by an expanse of time officially dedicated to developing research enabled me to create a comprehensive, multifaceted research design that was robust and flexible enough to bend and shift following the fundamental societal changes wrought by the ongoing pandemic. In developing what became a multilayered research design enabling me to more thoroughly explore the multifaceted research object of Indigenous radio, I realised how the Fellowship timeframe incorporated recognition that in-depth research planning needs to explicitly include dedicated time to build (as Mountz *et al.* argue) ‘community engagement [enabling] the pursuit of personally and politically meaningful work’ ( Mountz *et al.*, 2015: 1244). Before the Fellowship, like most researchers, my conceptualization, initial design and initial outreach to possible research participants was previously sandwiched alongside all my other time-specific academic responsibilities such as teaching, teaching preparation and related administration, standalone administrative duties, ongoing logistical arrangements and regular participation in staff meetings and discussions. As my mapping of Indigenous theory and Indigenous stations progressed and I began reaching out to possible research participants, I further realised that this temporal space also allowed for the necessarily gradual development of research relationships with participating practitioners; this was the more essential as relationships rightly take time to develop. To take one example, the station manager at Chickasaw station KCNP, introduced to me via my research collaborator at the University of Oklahoma, in turn introduced me to further Indigenous radio practitioners he regularly worked with. My Fellowship research timeframe provided time ‘accounted for planning and engagement, for following through’ ( Mountz *et al.*, 2015: 1245) which is essential for all relationship building. This greater temporal capacity also enabled me to more meaningfully incorporate the Indigenous principle of reciprocity, first discovered in my literature review of Indigenous critical theory and research methodologies. A central research aim of such reciprocity is to amplify Indigenous practice—not only those radio practices which are my research focus, but also raising awareness of a wider ‘development of Indigenous theory and methods of practice’ which, in turn, could ‘encourage a greater appreciation of Indigenous history and worldviews, thus allowing Indigenous peoples to look towards the future while neither demonizing nor romanticizing the past’, as Shawn Wilson suggests ( Wilson, 2008: 19). I attempt greater reciprocity in part through a practice of sharing all articles with practitioners for their feedback and editorial input in advance of publisher-mandated revisions. This practice enables me to check with those radio practitioners whose perspectives are represented that I have described their insights and work accurately and done justice to its importance for the communities they represent. It also enables practitioners to suggest their own clarifications which strengthen the article, making its arguments more robust by incorporating practitioner reflections alongside my own.

The continuing impact of the pandemic on Indigenous radio, and on community-led radio more broadly, remains a rich area for future study, albeit in continuing unsettled times. At the same time, as historic Indigenous land rights alongside ongoing fallout from historic institutional exploitation, discrimination and systemic abuses finally begin to be publicly negotiated and redressed in North American contexts, a long-delayed federal recognition of Indigenous self-determination is emerging which has been a long time coming following settler colonization. How this then impacts on Indigenous radio—and resources allocated—also remains to be witnessed.

By being apart, we’re now closer, at a different level, I think. I definitely think so.…We’ve learned a lot of lessons on how to communicate with people, that before wasn’t the normal way, and I think we’ll continue a lot of that. Through radio.

                                                                                                                                                                                                                          (Brashier interview, 2021)

As a radio scholar I see positive opportunities for Indigenous radio as the fundamental value of this form of community-building becomes more widely recognised; as a settler scholar writing from within a settler coloniser paradigm, I look forward to continuing to incorporate Indigenous critical theory in my research, both when exploring Indigenous radio and as part of a wider decolonising project further into the future.

## Data availability

### Underlying data

Full transcripts of interviews cannot be shared as participants did not consent to their publication.

Zenodo: Research Avenues for Amplifying Indigenous Radio. https://doi.org/10.5281/zenodo.5799427. ( Moylan, 2021, Research Avenues)

This project contains the following underlying data:

- IndigenousCriticalTheoryReadings.pdf (literature review)
- ListStationSchedules.pdf (list of radio station schedules)

This project contains the following extended data:

- ResearchStatementKMoylan843645.pdf (further information on ethics and consent)

Data are available under the terms of the Creative Commons Attribution 4.0 International license (CC-BY 4.0).

## Ethics and consent

Please note that all respondents whose insights are included have been informed about the nature of the overall project, Tribal Representation in Radio. Respondents have consented to full interviews and my recording of these, with the understanding that their responses may be included in research articles, following the established ethical approval protocols of the University of Leicester and approved in accordance with my EU Marie Skłodowska-Curie Global Research Fellowship. Participants did not consent to the publication of the full interview transcripts. In addition, I have disseminated pre-publication drafts to all respondents whose responses are incorporated in this article and within all other publications emerging from my research into Indigenous radio enabled by the Fellowship, for their approval, comments and any additional feedback.

## Footnotes

^1^ The wider estimate is arrived at drawing on information from Native Public Media, Native Voice One and the Native American Journalists’ Association (NAJA) and on anecdotal estimates from tribal radio practitioners, in conversation, as of January 2021. The figure given by Loris Taylor is cited in Denetclaw, P, 'Inside the Studio at KNON, Dallas’ Indigenous Radio Station', *Texas Observer*, 18 October 2021, https://www.texasobserver.org/indigenous-radio-in-texas/?fbclid=IwAR3t8qNIqzOO3WqcrakVqa7XVZidN9Py4_DC_pGcXTSXSqO8B1rAqfyyWjw, accessed 19 October 2021.

^2^ This figure from National Congress of American Indians, accessed via https://www.ncai.org/about-tribes, June 2021.

^3^ From KWLP’s homepage, http://kwlpradio.com/index.php, accessed 20 July 2021.

^4^ Description from *Native Noon Hour* show webpage, http://kwlpradio.com/index.php/radio-shows/cultural-native/native-noon-hour, accessed 20 July 2021.

^5^ See details of KWLP’s programmes at http://kwlpradio.com/index.php/station-info/daily-schedule, accessed 20 July 2021. Additional clarification provided by email communication from Terri Hutchens, 18 September 2021.

^6^ See details of KUYI’s programming at https://www.kuyi.net/program-schedule, accessed 20 July 2021.

^7^ Eric Ortega kindly wrote out the original Pala language saying for me during my visit to KPRI, 12 March 2020.

^8^ Information from KSUT Tribal Radio website at https://tribalradio.org/, accessed 22 July 2021.

^9^ Information from the KCNP/Chickasaw Network website, http://kcnp.org/, accessed 22 July 2021.

^10^ Further clarification provided by Brian Brashier via email, 17 September 2021.
