# Supplementary material for: Research avenues for amplifying Indigenous radio
Source: Open Res Eur. 2022 Mar 4;2:31. [Version 1] doi: 10.12688/openreseurope.14020.1 (PMC10445820; doi:10.12688/openreseurope.14020.1)
Supplement: Peer review history

# Peer review: Reviewer response for version 1

**DOI:** 10.21956/openreseurope.15108.r28713

**Published:** 2022-03-31

**Authors:** Caroline Mitchell

**Affiliations:** 1 Sunderland University, Sunderland, UK

Thank you for involving me in this review process, the first such open research review I have done. I have thoroughly enjoyed reading this study which I believe makes a major contribution to understanding community radio as a form of indigenous cultural expression and clearly communicates new avenues to help decolonise research/methods involving indigenous radio stations and practitioners.

 This comes from analysis of rich case study material developed through a multistranded research approach to indigenous radio.  Strengths include:

1. Development of participatory research work with participants from indigenous radio stations, addressing the need for sensitive and long-term methods to take into consideration conditions where these research participants are concurrently dealing with major events themselves, including running radio stations as part of survival infrastructure for extreme weather and pandemic conditions.
2. The (critical and counter) mapping of US indigenous radio stations as integral part of research through your Sovereignty of the Air: Amplifying Indigenous Radio Map. This is in itself a decolonizing research practice and tool.
3. For readers new to radio studies/community radio and US indigenous radio/public radio that incorporates indigenous programming, the article starts by explaining different categories of radio in the indigenous radio landscape and incorporates specialist language about radio station management and programming practices in an accessible way. Application of concepts such as ‘grounded normativity’; radio as an ‘everyday act of resurgence’, and ‘daily acts of renewal’ contribute to further understanding of new avenues relating to decolonising radio research practices and broadens language for discussing such practices.
4. Development of participatory research practices in pandemic conditions e.g. longer time to develop research relationships with radio station interviewees; caring and reflexive approaches to distanced research with people who are already providing front line services. This adds to currently limited research about the important role of localised community radio stations in the pandemic which apart from Coleman's early pandemic research in UK (2020) ^1^ has been limited to informal industry and journalistic accounts.

Minor points for consideration

1. You mention ‘Indianness’ of radio stations. Can this be linked to your useful concept of ‘accented’ radio that you’ve explored in previous studies?
2. There are several points where extreme weather is cited, e.g. ‘ because of the fires’ (Ortega interview quote) and I wonder if more contextual material can be written earlier about extreme weather in this part of USA and in the conclusion a mention of implications for research about climate justice and role of community/indigenous media in community building?
3. Sovereignty of the Air: Amplifying Indigenous Radio Map – would it be possible to embed links of station names for the case studies direct to the map?

Mapping: This is not central to this article but I was pleased to see the suggestion that your map could be further developed to include other nations and regions that have indigenous radio stations. I was wondering whether radio.garden currently contained these stations and whether there might be a themed intervention with radio.garden  to make sure this happened and possibly the recently added radio garden ‘playlist’ feature could be employed to highlight these stations

If applicable, is the statistical analysis and its interpretation appropriate?

Partly

Is the study design appropriate and is the work technically sound?

Yes

Is the work clearly and accurately presented and does it engage with the current literature?

Yes

Are the conclusions drawn adequately supported by the results?

Yes

Are sufficient details of methods and analysis provided to allow replication by others?

Yes

Are all the source data and materials underlying the results available?

Yes

Reviewer Expertise:

Community radio studies, transnational radio, community radio management and programming practices

I confirm that I have read this submission and believe that I have an appropriate level of expertise to confirm that it is of an acceptable scientific standard.

## References

[1] : UK Community Radio Response to COVID-19. *Brunel Univeristy of London* .2020; Reference source

# Peer review: Reviewer response for version 1

**DOI:** 10.21956/openreseurope.15108.r28711

**Published:** 2022-03-23

**Authors:** Paul Long

**Affiliations:** 1 Monash University, Melbourne, Australia

*Research avenues for amplifying Indigenous radio *relates in systematic fashion, some of the original and innovative findings of an EU Marie Skłodowska-Curie Global Research Fellowship in which the researcher explored the role of radio to Indigenous communities in the US. The focus is on 'Indigenous radio', that is those institutions in which tribal communities have control over constitution, production and content. 

 The research provides substantive insights into the character and practices of several tribal stations which underlines the trade-off between the holistic approach to this 'sector' and the specificities of local cultures.

 Methodologically, the research is sensitive the implications of both mapping and close attention to the worlds depicted and the power dynamics of extant investigative and theoretical paradigms. The empirical work has been robustly conducted and is conveyed meaningfully. drawing upon interviews and analysis of schedules and content. Supporting documents are indicated but restricted according to ethical principles.

 The scholarly context is pithily outlined, attuned to Indigenous theorists and theories which are usefully employed e.g. 'grounded normativity'. Other readings and contexts would reveal analogous formulations and ideas although the rationale for inspiration and deployment is well rationalised and empathetic.

 Overall the article is clearly organised and written in an accessible manner without any loss of sophistication. The insights into the value of radio for communities in general and in light of Covid impacts are meaningful and deserving of wider attention and comparison. 

 In scope and scale, the piece satisfies in its current length although a number of questions arise about items that might merit some comment or that one hopes will be taken up in further work. 

 An obvious question alludes to the responsiveness of audiences, intended or otherwise, to station output and identity. We gain insights from the producers' perspective about value and impact but little from the constituency and the choices they make for attention. In the US context and the stations described one wonders at any obligations to know the audience officially or otherwise. The element is an important component of the practices described here - for those in the tribal communities as well as those 'outside' who be addressed directly or indirectly.

 There is a narrative element to the research reported and resultant theorisation in which the author relates her own trajectory in the fellowship (listed in prelims etc, although perhaps worth signalling in prose in the main text) is sensitive to her status as 'settler scholar' writing from within a situated paradigm. This status and perspective properly inflect the reporting of relations with research subjects although given this framing it would be useful to hear about any tensions attendant upon this position which go unremarked. For instance, there are ongoing debates about representational politics and who is empowered to speak on whose behalf and about marginalised communities which is worthy of reflection as method and theory. I'm sure the author is sensitive to such issues and one would like to know in some small measure what is 'at stake' for her. For instance, from the perspective of being a radio scholar (a relatively minor pursuit), it is valuable to read an original contribution of such impactful practices for US Indigenous communities that would have value elsewhere and which emphasises the importance of this field. Nonetheless, and given the aforementioned sensitivities, there's more to be said here.

 On an empirical note, one thing that this reader wanted to know about is the extent to which any of the programming and practices described reproduces local languages. One snippet of *Pala *language is given and translated on p.7 but there is no other reference to whether or not stations are uni or multi-lingual.

 On a technical note, we have some sense of the footprint of broadcast and its relationship to geography and locality but also the reach of stations thanks to the digital (which also afforded this reviewer a chance to listen in to the objects of study, albeit briefly). I think there's something more complex to be teased out here but again, for another piece perhaps.

 Use is made of Gill and Pratt writing on cultural work and immaterial labour. This is perhaps a little superficial as is and merits some remark for the challenges this kind of research and practice poses for the absences of the paradigms that have emerged around such studies around labour, value, etc. 

 On a cultural note, and there is perhaps no space in the article for this, the variety of musical programming is also worthy of remark and reference to the choices made about what kinds of relations exist with popular cultural forms such as reggae. The prism of popular culture is an interesting one to consider the very real challenges of conceptualising the integrity of Indigenous cultures and an understandable tendency to partition them off from 'modernity' and its impacts as resulting from colonisation when there are also inputs, inspirations and appropriations to consider.

If applicable, is the statistical analysis and its interpretation appropriate?

Yes

Is the study design appropriate and is the work technically sound?

Yes

Is the work clearly and accurately presented and does it engage with the current literature?

Yes

Are the conclusions drawn adequately supported by the results?

Yes

Are sufficient details of methods and analysis provided to allow replication by others?

Yes

Are all the source data and materials underlying the results available?

Partly

Reviewer Expertise:

Cultural and creative industries;  media studies; cultural heritage.

I confirm that I have read this submission and believe that I have an appropriate level of expertise to confirm that it is of an acceptable scientific standard.
